# Health Tract No. 335

**Published:** 1868-09

**Authors:** 


HEALTH TRACT NO. 335.

### EYESIGHT.

At the age of seventy years a name honored and revered on both continents writes, "I am now writing this with my eyes closed, by the aid of a machine and even this at some peril of blindness. My general health is perfect, and I am able to do as much work as ever, without fatigue. My only difficulty is with my eyes and this is a serious and alarming one." To have good health, and to be capable both as to mind and body of doing full work, and yet not be allowed to do any, and this to have been the case, more or less, for ten years past, and to last for all the life to come, as it certainly will, is a terrible calamity; a clear loss of twenty years labor to the world. This condition was induced by the person getting up to study and write at four o'clock winter and summer for a series of years. A beneficient Providence has arranged that the glare of light shall come on very gradually in the morning and that as gradually shall it depart into darkness in the evening. The painfulness of coming instantly into a bright light is familiar to all. And yet after the eyes have been closed in the perfect darkness of sleep for seven or eight hours, to be instantly exposed to a bright gas or other artificial light, for early study is practised by many; and without knowing it very many students thus prepare themselves for an early impairment of sight, to say nothing of the bodily suffering, of mental chafing and disquietude and loss of time and money. There is no gain, in the long run by using the eyes to read or write after sundown or before sunrise and breakfast; it may be done with a measure of impunity in a few cases; but in nine cases out of ten disaster will follow; in no case is night study an economy of time, nor is it a necessity as a habitual thing. Night is the time for rest, and both body and brain, especially as to students, require all the sleep the system will take; they ought never to be waked up, nature will infallibly do that when she has had her fill, and to shorten sleep, is to shorten life; half the time of daylight is as long as any man ought to spend in hard study.

